# Vimig: The virus-induced migrasome as a novel mechanism for viral transmission and communication

**DOI:** 10.1371/journal.ppat.1013557

**Published:** 2025-10-09

**Authors:** Mingyan Feng, Leiliang Zhang

**Affiliations:** 1 Department of Clinical Laboratory Medicine, The First Affiliated Hospital of Shandong First Medical University & Shandong Provincial Qianfoshan Hospital, Jinan, Shandong, China; 2 Department of Pathogen Biology, School of Clinical and Basic Medical Sciences, Shandong First Medical University & Shandong Academy of Medical Sciences, Jinan, Shandong, China; University of Iowa, UNITED STATES OF AMERICA

## Abstract

Vimig, defined as “virus-induced migrasome,” represents a novel class of extracellular vesicles that originate from virus-infected cells. The mechanisms underlying vimig formation involve actin remodeling and upregulation of phosphatidylinositol (4,5)-bisphosphate (PI(4,5)P2). Vimig not only encapsulates viral particles but also aids in the transport of damaged organelles, including mitochondria, thereby contributing to cellular homeostasis and potentially enhancing viral spread and infection. Characterized by their unique contents, which includes viral particles, lipids, proteins, and cellular debris, vimig serves as a transmission route for viruses, possibly allowing them to evade host immune responses. This pearl summarizes the biogenesis, functional significance, and implications of vimig in viral pathogenesis, emphasizing its potential as a target for therapeutic interventions aimed at mitigating viral infections. Understanding the role of vimig may pave the way for novel strategies in clinical drug development and deepen our insights into virus–host interactions.

**Q: What is vimig?**
**A:** We define vimig as the abbreviation of “virus-induced migrasome”. As early as 2014, Dr. Li Yu’s team reported a “pomegranate-like structure” related to cell migration in the extracellular space around rat kidney cells and named it “migrasome” (Fig 1) [1]. During cell migration, cells dynamically interact with their environment, forming specialized structures at the leading and trailing edges of the migrating cell, namely, retraction fibers (RFs) identified in 1963 (Fig 1) [2]. These unique extracellular vesicles, ranging from 0.5 to 3 μm in diameter, located at the branches or tips of RFs, being released at the trailing edge during cell migration and function as a tool of intercellular communication [1,3]. Since being discovered, migrasome has attracted considerable research attention as a new type of subcellular structure due to its unique biogenesis, structural characteristics, and essential functions in various biological processes. Interestingly, migrasome could be induced by several viruses, including severe acute respiratory syndrome coronavirus 2 (SARS-CoV-2) [4], chikungunya virus (CHIKV) [5], vaccinia virus (VACV) [6], and herpes simplex virus 2 (HSV-2) (Fig 1) [7]. Therefore, we propose a new concept of “vimig” to describe the specific migrasome that arises in the virus-induced environment, to better understand the relationship between migrasome and host interactions in virology.
**Q: How do the viruses induce the biogenesis of vimig?**
A: The formation of migrasomes is intricately linked to both cell migration and actin polymerization. As migrating cells advance, actin remodeling produces RFs that extend at the posterior part of the cell, where migrasomes are generated [1]. Initially, sphingomyelin synthase 2 (SMS2) assembles into fixed foci and attaches to the basement membrane at the leading edge of the cell [8]. As the cell migrates, the foci formed by SMS2 move out of the cell and enter the RFs, where they function as the site for migrasome formation. Meanwhile, phosphatidylinositol (4,5)-bisphosphate (PI(4,5)P2)‐Rab35 axis plays a significant role in the regulation of migrasome formation and localization, with integrin α5β1 serving as a key molecule for anchoring the migrasome to the extracellular matrix [9]. Furthermore, the recruitment of tetraspanin 4 (TSPAN4) and cholesterol facilitates the rapid formation and stabilization of migrasomes [10,11].
Vimigs have been morphologically observed in cells infected with CHIKV, SARS-CoV-2, VACV, and HSV-2 [4–7]. Investigation into vimig remains incomplete, particularly concerning its size and quantity. While VACV-induced vimig is reported to range from 1 to 3 μm [6], the sizes of vimig induced by CHIKV, SARS-CoV-2, and HSV-2 are not precisely documented. Unlike exosomes, which are released through the fusion of multivesicular body with the plasma membrane, migrasomes are released via the breakage of RFs. This biological process requires the involvement of actin [1,3]. Intriguingly, vimig can be directly initiated by viral protein [5]. The transfection of a plasmid expressing Flag-tagged CHIKV-nsP1 can directly induce the formation of migrasomes. Further research implicated that nsP1 could recruit and activate PIP5K1A to enhance the production of PI(4,5)P2, promoting CHIKV replication and vimig formation. Furthermore, the nsP1 protein of CHIKV could not only induce vimig formation but also facilitate cell migration [5]. While the viral nsP1 protein of CHIKV can directly trigger vimig formation, it can also do so through the interactions with the key molecules as aforementioned, which means the vimig generation cannot be fully independent of the process of general migrasome formation. In addition, vimig has also been observed in poxviruses and HSV-2 infected cells, with an unclear mechanism [6,7]. Taken together, it is likely that poxvirus and HSV-2 may facilitate the generation of vimigs by interacting with key proteins or lipids involved in the known regulatory axis of migrasome formation.
**Q: What are the contents of vimig?**
A: Just like general migrasome, vimig serves as the main tool for migracytosis, a unique form of cellular communication that involves the packaging and transfer of proteins, lipids, and RNA (Fig 2) [12]. Additionally, viral particles and other nanoscale small molecules, such as polystyrene nanoparticles (PS-NPs), have been observed in vimigs under specific circumstances. Our work is the first to identify the presence of virions within vimig induced by viral infection. Using VACV as a model virus for poxvirus, we observed vimig ranging from 1 to 3 μm during the late stage of infection. More specifically, the virions encapsulated by the migrasomes are in intracellular mature virus (IMV) and intracellular enveloped virus (IEV), which are the two infectious virions of poxviruses [6]. Intriguingly, PS-NPs can be involved in the regulation of vimig [13]. The presence of PS-NPs notably speeds up the entry of VACV into vimig, altering how the virus spreads. Furthermore, this study also suggested that VACV infection resulted in the transportation of lipid droplets to vimig, thereby enhancing its functional capacity. PS-NPs can enhance the size of intracellular lipid droplets more efficiently than a solo VACV infection by upregulating the levels of PI(4,5)P2 and cholesterol [13]. Furthermore, HSV-2 virions were found in the contents of the isolated migrasomes [7]. Additionally, the presence of CHIKV virions within vimig was revealed [14]. Therefore, the contents of the vimig are much more variable than those of the general migrasomes, which may involve the interaction of some exogenous small molecules with viral proteins and host proteins.
**Q: What is the biological function of vimig?**
A: Considering the role of migrasomes in mediating cell-to-cell communication and the presence of virions within vimig, we propose that the primary biological function of vimig is to facilitate viral transmission and infection (Fig 2). It has been discovered that VACV-induced vimigs contain virions, including both IMV and IEV [6]. The poxvirus F13 protein is found in IEV and extracellular enveloped virus but not in IMV, which is the target of the anti-poxvirus drug tecovirimat (also known as ST-246 or TPOXX). Tecovirimat is a small molecule developed to combat poxvirus infections and is recognized for its balanced antiviral efficacy, metabolic stability, and low toxicity [15]. However, in clinical settings, tecovirimat appears to have a low resistance barrier [15]. Additionally, it has failed to effectively treat mpox due to IMV escape [16]. Considering the role of IMV, these findings suggest that vimig may represent a novel route for poxvirus spread, potentially contributing to resistance against tecovirimat treatment. Furthermore, purified vimigs generated by HSV-2-infected HaCaT cells, rather than the whole infected cells, can lead to the infection of both HSV-2 permissive cells (HaCaT) and non-permissive cells (CHO) [7]. This suggests a novel mechanism of HSV-2 cell-to-cell spread mediated by vimigs. Traditionally, CHIKV is considered to be assembled and released from the plasma membrane. However, CHIKV virions locate not only in the plasma membrane but also inside vimig, demonstrating a new exit route for CHIKV. Meanwhile, different from receptor-mediated virus entry, vimig may help CHIKV escape from neutralizing antibodies, emerging as a novel mode of viral transmission that protects virions against antibody neutralization. Notably, the vimig generated by SARS-CoV-2-infected platelets contains cytokines and play a role in mediation of cell-cell communication, contributing to dysregulated immunity and thrombosis (Fig 2) [4].
**Q: Is there anything else that needs to be discussed of vimig?**
A: Firstly, vimig, defined as migrasome generated by virus-infected cells, has been discovered only in association with four types of viruses (SARS-CoV-2, CHIKV, VACV, and HSV-2) [4–7], which suggests a potential mechanism inhibiting vimig formation to be elucidated. Notably, any blockage of pathways involved in migrasome generation, such as SMS2 inhibition or a lack of cholesterol, could lead to the absence of vimig.
Secondly, studies have shown that faster, more directed cell movement leads to RFs and more frequent migrasome formation at the RF ends [17]. Meanwhile, CHIKV nsP1-transfected cells migrated faster in scratch test, suggesting that nsP1 facilitated cell migration [5]. However, no studies have shown how much cell migration plays a role in the production of vimig.
Thirdly, in cells undergoing migration, which experiences heightened mitochondrial stress due to increased energy demands, damaged mitochondria are selectively transported to the cell’s periphery and sequestered into migrasomes [18] In the context of viral infection, the surging energy demands and direct lesion caused by virus infection may also lead to the transportation of damaged mitochondria or other organelles including lysosomes into vimig (Fig 2).
Furthermore, vimig had been proposed as a potential transmission mode of VACV, HSV-2, and CHIKV [6,7,14]. These viruses may evade neutralizing antibodies by hiding in vimig. This alternative transmission route highlights the need for increased attention in clinical drug development and the formulation of targeted therapeutic strategies.

**Fig 1:**
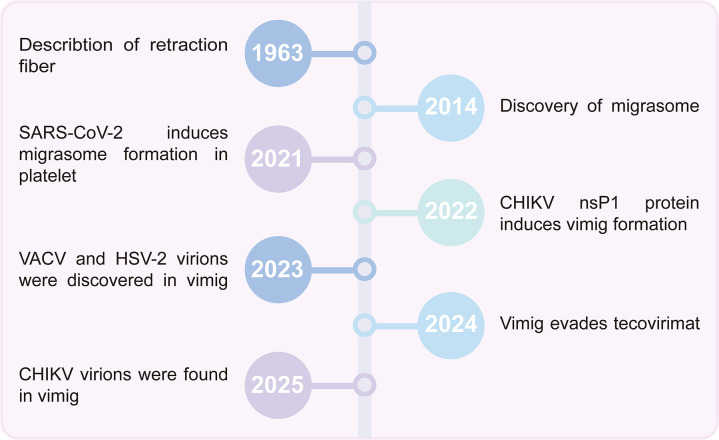
Key milestones in the discovery of vimig.

**Fig 2:**
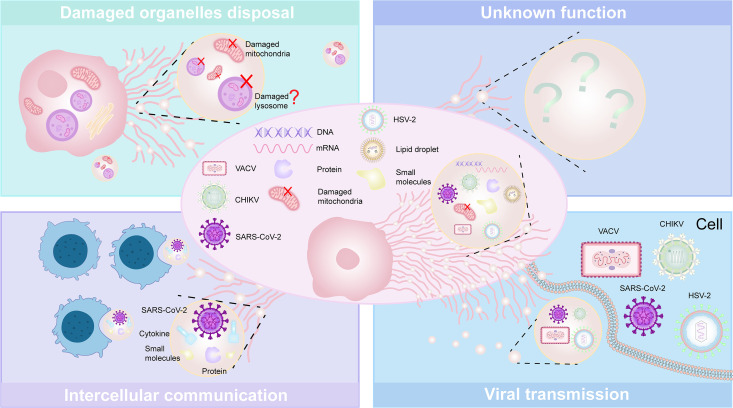
Main components and fundamental biological roles of vimig. Vimig contains DNA, RNA, proteins, lipids, damaged mitochondria, exogenous small molecules, and various virions including VACV, CHIKV, SARS-CoV-2, and HSV-2. Vimig selectively transports damaged mitochondria into the extracellular environment through a process known as mitocytosis. Other organelles, such as lysosomes, may undergo a similar process. SARS-CoV-2-induced vimig contains viral particles and other intercellular communication substances. Additionally, vimig represents a novel mode of viral transmission, involving poxvirus, CHIKV, SARS-CoV-2, and HSV-2, with the potential to facilitate viral infections by evading neutralizing antibodies.
